# Cyclin-Dependent Kinase 5 Is Involved in the Phosphorylation of Gephyrin and Clustering of GABA_A_ Receptors at Inhibitory Synapses of Hippocampal Neurons

**DOI:** 10.1371/journal.pone.0104256

**Published:** 2014-08-05

**Authors:** Heba Kalbouneh, Andrea Schlicksupp, Joachim Kirsch, Jochen Kuhse

**Affiliations:** Department of Anatomy and Cell Biology, University of Heidelberg, Heidelberg, Germany; University of Sydney, Australia

## Abstract

CDK5 has been implicated in neural functions including growth, neuronal migration, synaptic transmission and plasticity of excitatory chemical synapses. Here we report robust effects of CDK5 on phosphorylation of the postsynaptic scaffold protein gephyrin and clustering of inhibitory GABA_A_ receptors in hippocampal neurons. shRNA-mediated knockdown of CDK5 and pharmacological inhibition of cyclin-dependent kinases reduced phosphorylated gephyrin clusters and postsynaptic γ2-containing GABA_A_ receptors. Phosphorylation of S270 is antagonized by PP1/PP2a phosphatase and site-directed mutagenesis and *in vitro* phosphorylation experiments indicate that S270 is a putative CDK5 phosphorylation site of gephyrin. Our data suggest that CDK5 plays an essential role for the stability of gephyrin-dependent GABA_A_ receptor clusters in hippocampal neurons.

## Introduction

Cyclin-dependent kinase 5 is a proline-directed serine/threonine kinase belonging to the class of CDC2 (CDK1) -like kinases [1]. CDK5 activity is dependent on the association with neuron-specific regulatory proteins p35 or p39 [2], [3]. The calpain-dependent cleavage of p35 generates a CDK5 activator (p25) with distinct cellular localization and activity [4]. Interestingly, CDK5 has been implicated in a huge number of neuronal functions, brain development and diseases [5]. CDK5 activity was reported to be crucial for neuronal migration [6], cortex layer formation [7], neurite outgrowth [8], [9] and retrograde axonal transport [10]. Phosphorylation of various synaptic substrates was shown to have a major impact on pre- and postsynaptic functions of excitatory synapses. For example, analysis of the CDK5-dependent phosphorylation of PSD95 [11] implies a functional role of CDK5 for regulating synaptic strength by modulating postsynaptic localization and density of glutamate-gated ion channels. Moreover, different studies showed a CDK5-dependent change of postsynaptic NMDA receptor clustering depending on subunit phosphorylation [12]. Consistent with these reports, Li *et al*. showed that CDK5 inhibition blocks long-term potentiation (LTP) induction in CA1 hippocampal neurons [13]. Similarly, another study revealed that CDK5 loss-of-function in hippocampal circuits results in severe impairments in memory formation and retrival associated with LTP deficits in hippocampal CA1 neurons [14].

Whereas these and other studies suggest an involvement of CDK5 in the regulation of glutamatergic neurotransmission, almost nothing is known about a putative functional role of CDK5 at inhibitory synapses. We report that knockdown or inhibition of CDK5 in hippocampal neurons results in reduced phosphorylation of postsynaptic clusters of the scaffold protein gephyrin, detected with the phospho-specific antibody mAb7a [15]. Moreover, CDK5 knockdown or inhibition results in a reduced number of γ2 containing GABA_A_ receptor clusters, suggesting that CDK5 plays an important role at inhibitory synapses.

## Experimental Procedures

### Ethics statement

This study was carried out in strict accordance with the European Communities Council Directive (86/609/EEC) to minimize animal pain or discomfort. The local animal care and use committee (Interfakultäre Biomedizinische Forschungseinrichtung (IBF) Heidelberg) gave approval for the study under the number T-81/10.

### Construction of expression plasmids and knockdown vectors

DNA fragments encoding the complete coding sequence of rat CDK5 were amplified by PCR from embryonic brain cDNA creating flanking *Bam*HI and *Hind*III restriction sites and were cloned in the expression vector pCMVTAG 3B (Invitrogen) resulting in *N*-terminal fusion constructs with a myc-epitope (myc-CDK5). Sequences encoding for three shRNA directed against CDK5 and scrambled shRNA were cloned into pFSGW, a modified version of the lentiviral vector pFUGW [16], using a human synapsin promoter to drive EGFP expression. CDK5-kd1: 5′tttgaagctcacattggtgtttga3′, CDK5-kd2: 5′tttgaatctgctcattaacaggaa 3′, CDK5-kd3: 5′tttgaccaaactgccagactataa3′- and a control scrambled sequence (mismatch): 5′tttgtgtgcaaatattcagcgaa 3′.

### Site-directed mutagenesis

Site-directed mutagenesis of 6xHistidine-tagged gephyrin was performed using the Quickchange Lightning mutagenesis kit from Stratagene following the suppliers' instructions. Mutated sequences were verified by sequence analysis.

### Lentivirus preparation

Recombinant lentiviral particles were produced as described earlier [15] and references therein.

### Cell culture

Primary cultures of rat hippocampal neurons were prepared from E19 embryos plated at a density of 60.000 cells/cm^2^ as described previously [17]. Infection with lentivirus dilutions was performed six days after plating (div6). Fixation and immunocytochemistry was done at div14.

### Pharmacological treatment of cultured hippocampal neurons

Treatment of cells was done as described earlier [15].

### Immunocytochemistry

Immunocytochemistry was performed as described earlier [15]. For Ab-175 immunolabeling cultured neurons were washed once with PBS at 37°C and fixed with cold methanol (−20°C) for 10 min. Cells were incubated with the primary antibody dilutions of mouse monoclonal anti-gephyrin mAb7a [18] and rabbit anti-gephyrin Ab-175 [19] as described earlier [15]. Cells were incubated with the following additional antibodies: chicken anti GABA_A_R gamma2-subunit antibody (1∶10) (BD Transduction Labs, Cat. No.: 612076), rabbit anti-vesicular inhibitory amino acid transporter (VIAAT) antibody (1∶2000) (Sigma, No.: V 5764), rabbit anti-CDK5 antibody (1∶200) (Santa Cruz, No. sc-173). Analysis of antibody labeling was performed using an Axiovert 200M microscope (Zeiss) equipped with a SPOT RT CCD camera (Diagnostic Instruments, Sterling Heights, MI). Images were collected with a 40× or a 63× Plan-Apochromat (1.4 NA) objective (Zeiss). For quantifications MetaMorph and Fiji software were used. For quantification the fluorescence intensities of single synaptic puncta, mAb7a and Ab-175 fluorescence images were taken with a Leica confocal TCS SP8 microscope using a 63× Plan-Apochromat (1.4 NA) objective. To evaluate the number of gephyrin or GABA_A_ receptor clusters, one to three proximal dendritic regions with a length of 30 µm were chosen. For determining the number of synaptic gephyrin clusters VIAAT-fluorescence was used for region selection and consequently the respective postsynaptic immunofluorescence signals were analyzed. After setting the same threshold for these regions for all immunolabelings of one experiment, the quantification of gephyrin or γ2 puncta was determined by the software. Data analysis was done using Excel and Prism software.

### Gephyrin purification

N-terminaly 6xHistidine-tagged gephyrin wild type and 6xHistidine-tagged gephyrin-mutant proteins were expressed and purified as described elsewhere [15].

### *In vitro* ^32^P-phosphorylation assay

Immunoprecipitation of ^32^P-labeled gephyrin was done using the gephyrin-specific antibody mAb5 [18] that was prebound to proteinA/G agarose (Pierce, Thermo Scientific) at 4°C for 15 hrs. Identical amounts of purified 6xHis-gephyrin wild type or mutant proteins were used for *in vitro* phosphorylation assays performed in HEPES buffer with 30 µCi [γ-^32^P] ATP (3000 Ci/mmol) and 10 µM unlabeled ATP, 0.1 U CDK5/p25 GST protein complexes (Biaffin, Kassel, Germany) at 30°C for 30 min and further processed as described earlier [15]. Proteins were separated on 4–12% polyacrylamide gradient SDS-gels (NuPAGE Novex Bis-Tris mini gels, Invitrogen). Gels were stained with Coomassie blue as proof of equal protein loading, dried on a vacuum gel dryer and exposed to X-ray Kodak films.

### Statistical analysis

Experimental data were evaluated of at least three independent experiments without knowledge of the experimental conditions used. Statistical analysis was performed using the GraphPad-prism IV software with non-parametric ANOVA followed by Tukey's or Bonferroni's multiple comparison tests, other data were analyzed with Student's t-test. Data are shown as mean ± standard error of the mean (SE) or in the analysis of the in vitro ^32^P-phosphorylation assay as mean ± SD. Values of P<0.05 were considered as significant.

## Results

### Reduction of CDK5 expression in hippocampal neurons results in a reduced number of immunoreactive gephyrin puncta

To investigate the functional role of CDK5 for the phosphorylation of gephyrin clusters at GABAergic synapses of cultured hippocampal neurons, we first established a knockdown of CDK5 with three different shRNA expressing lentiviruses. After having established that these sequences efficiently reduced heterologously expressed, myc-tagged CDK5 in HEK293T cells (data not shown), the reduction of CDK5 expression by these shRNAs in cultured hippocampal neurons was analyzed by immunofluorescence microscopy. As control, a scrambled shRNA (mismatch) was used.

**Figure 1:**
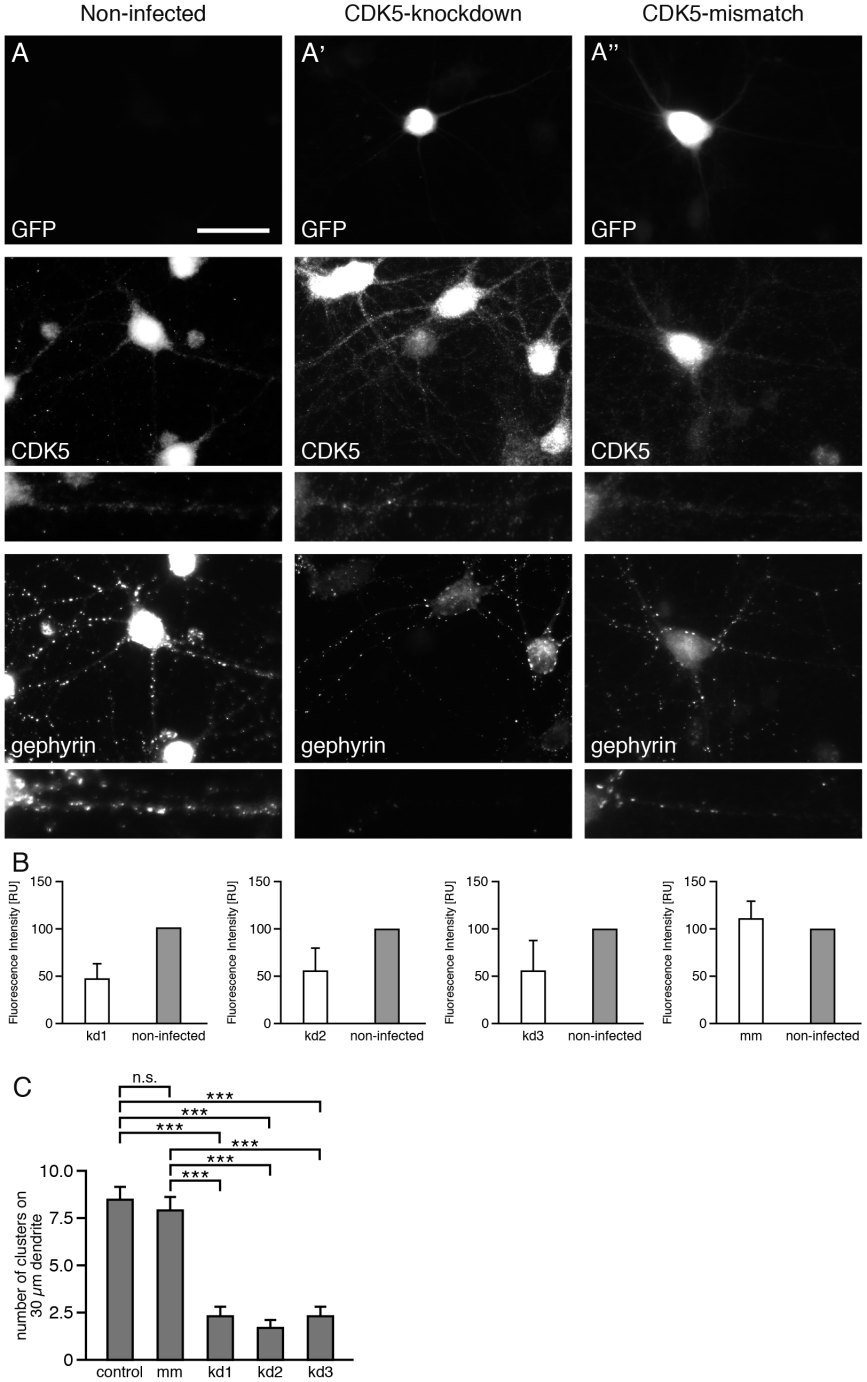
CDK5 knockdown virus infection results in reduced CDK5 expression and reduced numbers of phospho-gephyrin clusters in cultured hippocampal neurons. Hippocampal neurons (div14) were stained with anti-GFP antibodies to detect infected neurons (upper panel), with anti-CDK5 antibody to quantify CDK5 expression levels (middle panel) and with the phosphospecific anti-gephyrin mAb7a antibody (lower panel). (A) Non-infected cells; (A') CDK5-knockdown; (A'') control shRNA (CDK5-mismatch). Neurons were infected with the indicated viruses at div6. The scale bar represents 15 µm. (B) Quantification of CDK5 fluorescence intensities of neurons infected with three different CDK5 knockdown viruses (kd1, kd2 and kd3). CDK5 knockdown cells were compared to non-infected neurons. n = 3, mean ± SD (C) Quantification of mAb7a cluster numbers of hippocampal neurons infected with three different CDK5 knockdown viruses (kd1, kd2, kd3) compared to non-infected and control shRNA (mismatch). 30 cells from n = 4 independent cultures (for each of non-infected, mismatch, kd2, kd1) or 30 cells from n = 3 independent cultures (kd3), mean ± SE, ANOVA with post-hoc test, ***P<0.001.

Cells infected by the CDK5-kd viruses - as indicated by GFP expression (Fig. 1) - revealed about 50% reduction in CDK5 expression (Fig. 1B). Neurons with reduced CDK5 expression (Fig. 1A') showed a robust reduction of phosphorylated gephyrin puncta (Fig. 1C), as demonstrated with the phospho-specific antibody mAb7a [15], whereas a control shRNA (Fig. 1A'') did neither reduce CDK5 expression nor mAb7a staining of the infected cells. The quantitative analysis of mAb7a-specific immunoreactive puncta of three independent CDK5-specific shRNAs revealed a comparable reduction of gephyrin puncta with kd1 (knockdown1): 2.2±0.77 puncta/30 µm (mean ± SE), 1.58±0.43 puncta (mean ± SE) for kd2, and 2.24±0.39 puncta (mean ± SE) for kd3 compared to non-infected neurons: 8.47±0.74 puncta, or scrambled control shRNA (mm): 7.8±0.74 puncta, each per 30 µm dendrite (Fig. 1C). These data support the conclusion, that the CDK5 knockdown is functionally linked to a reduction in gephyrin cluster phosphorylation.

### Reduction of CDK5 expression results in a reduced number of GABA_A_ receptor γ2 clusters

**Figure 2:**
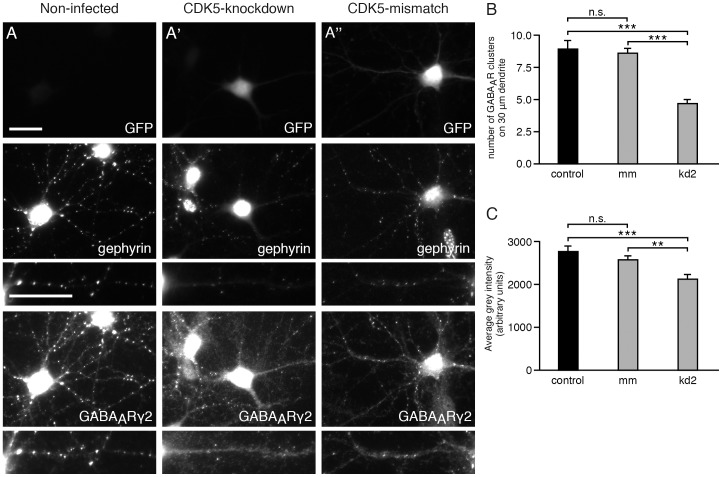
CDK5 knockdown correlates with reduced numbers of GABA_A_ receptor clusters containing γ2-subunits. Hippocampal neurons (div14) were immunolabeled with anti GFP antibodies to detect infected neurons (upper panel), with phosphospecific anti gephyrin mAb7a antibody (middle panel), and with anti-GABA_A_ receptors γ2-subunit of (lower panel). (A) Non-infected cells; (A') CDK5-kd2 knockdown; (A'') control shRNA (CDK5-mismatch). Neurons were infected with the indicated viruses at div6. The scale bar represents 15 µm. (B) Quantification of the number of GABA_A_ receptor γ2 puncta on three proximal dendritic segments of 30 µm. 20 or 18 cells from n = 3 independent cultures of control or mismatch neurons and 25 cells (69 dendrites) from n = 4 independent cultures of kd2-infected neurons, mean ± SE. ANOVA with post-hoc test, *** P<0.001. (C) Quantification of the relative fluorescence intensities of GABA_A_ receptor γ2 puncta. Mean ± SE. Each value was calculated from data from the same cell numbers as in B from n = 3 independent cultures. ANOVA with post-hoc test: *** P<0.001, ** P<0.01.

To determine whether reduced number of phosphorylated gephyrin clusters detected with mAb7a at postsynaptic sites could affect the clustering of endogenous GABA_A_ receptors, the subcellular localization of GABA_A_ receptor γ2 subunits was investigated. GABA_A_ receptors harboring this subunit are known to colocalize with and to depend on gephyrin for synaptic localization in hippocampal neurons [20], [21]. As shown in Fig. 2A and 2B, infection of cultured hippocampal neurons with CDK5-kd2 resulted in a strong reduction of mAb7a-immunoreactive puncta. In addition, a reduction of GABA_A_ receptor γ2-immunoreactive puncta along dendrites could be seen, whereas the immunoreactivity within the soma was preserved. The quantification shows that the number of GABA_A_ receptor γ2 clusters is significantly reduced (Fig. 2B) kd2: 4.64±0.38 puncta/30 µm (mean ± SE) compared to 8.54±0.41 (mean ± SE) puncta for control shRNA (mm) and 9.67±0.45 puncta (mean ± SE) for non-infected hippocampal neurons. Moreover, the average fluorescence intensities of clusters were reduced upon CDK5 knockdown (Fig. 2C). Thus, a knockdown of CDK5 results in a reduced number and density of γ2-containing GABA_A_ receptor clusters in hippocampal neurons.

**Figure 3:**
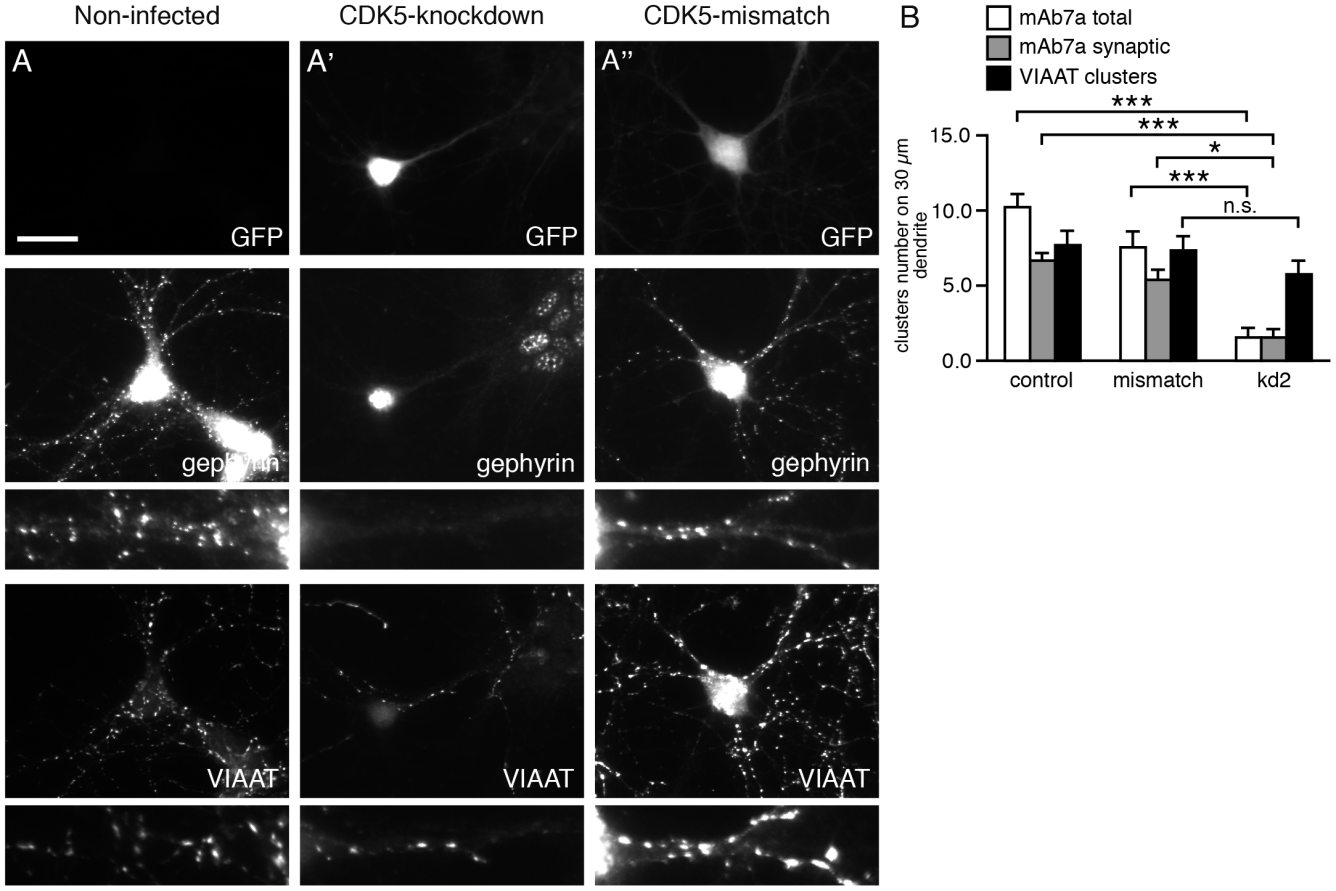
Synaptic mAb7a-specific gephyrin clusters are reduced upon CDK5 knockdown whereas VIAAT puncta were not reduced in perisomatic dendritic segments. Hippocampal neurons (div14) were immunolabeled with anti-GFP antibodies to detect infected neurons (upper panel), with phosphospecific anti-gephyrin mAb7a antibody (middle panel), and with anti-VIAAT antibody (lower panel). (A) Non-infected cells; (A') CDK5-kd2 knockdown; (A'') control shRNA (CDK5-mismatch). Neurons were infected with the indicated viruses at div6. The scale bar represents 15 µm. (B) Quantification of the number of VIAAT puncta, mAb7a puncta and overlapping mAb7a/VIAAT puncta (synaptic mAb7a clusters). Mean ± SE. Each value was calculated from analysis of 15 cells from n = 3 independent cultures, ANOVA with post-hoc test: *** P< 0.001, * P<0.05.

As a control for the specificity of the effect of CDK5 knockdown on GABA_A_ recepto cluster number and density the immunofluorescence of the presynaptic marker vesicular inhibitory amino acid transporter (VIAAT) was analyzed. Although a small reduction of the number of VIAAT puncta could be observed in kd2 infected cells (Fig. 3A and B), the VIAAT puncta showed no significant changes within the proximal dendrites analyzed (Fig. 3B). The VIAAT and mAb7a double staining was also used to determine the number of VIAAT-apposed mAb7a-positive gephyrin clusters. As shown in Fig. 3, the CDK5 knockdown reduced the number of synaptic mAb7a puncta above a certain intensity threshold drastically. Hence, we conclude that CDK5 is specifically involved in the phosphorylation of synaptic gephyrin clusters.

### *CDK5 inhibition results in a reduced number of* mAb7a-positive gephyrin clusters and GABA_A_ receptor γ2 clusters

**Figure 4:**
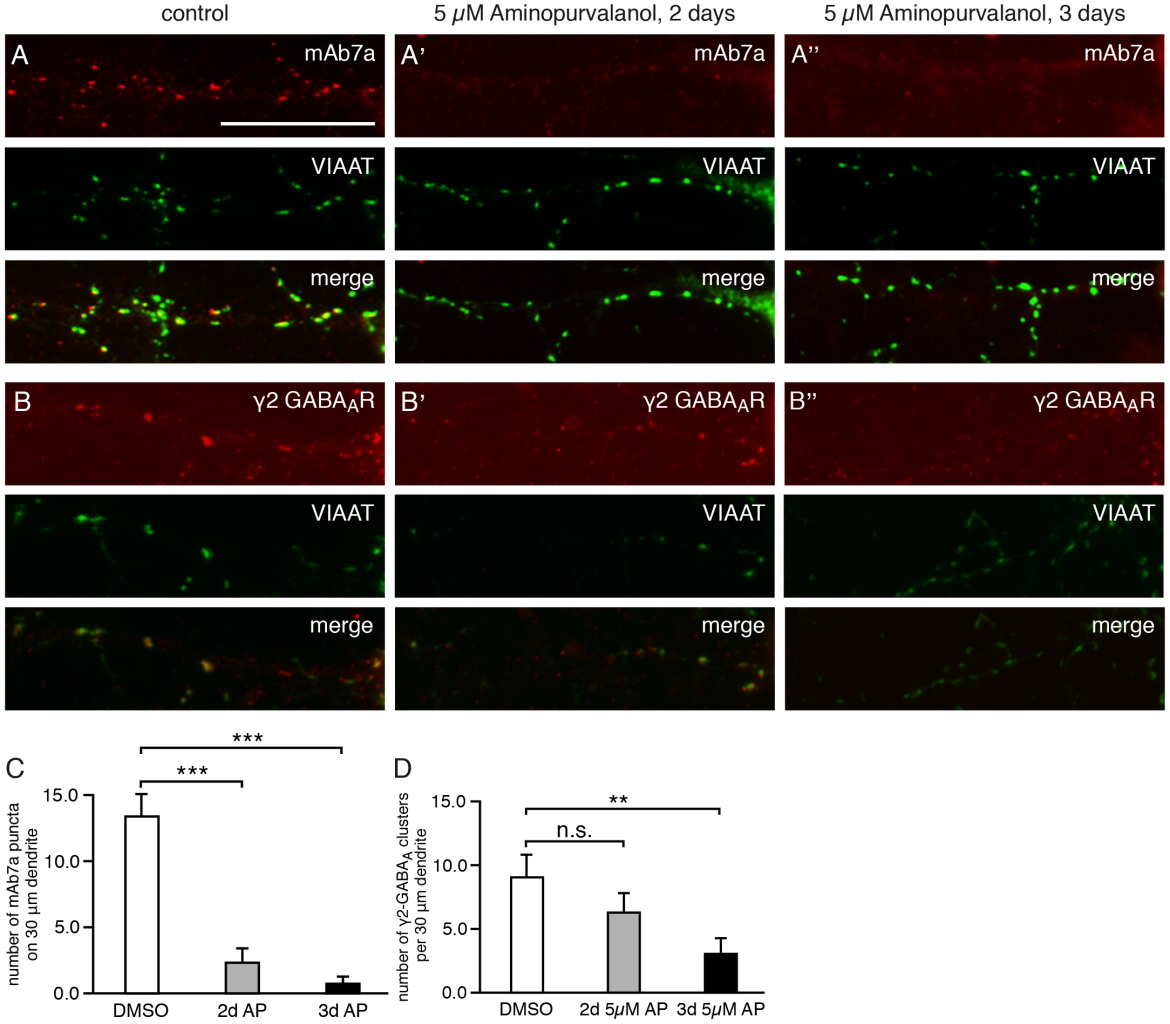
Gephyrin and GABA_A_ receptor γ2 puncta are reduced upon CDK5/2/1 inhibition. (A) Hippocampal neurons were double-immunolabeled for gephyrin with antibody mAb7a (red, upper panel) and with anti-VIAAT antibody (green, middle panel). Superposition of both immunolabelings (lower panel, merge). Neurons were fixed and immunolabeled at div16. (A) Control cells, non-treated; (A') cultures treated with aminopurvalanol A (5 µM) for 2 days; (A'') cultures treated with aminopurvalanol A (5 µM) for 3 days. Note a higher number of VIAAT-opposed gephyrin puncta (yellow, merge) under A, compared to A' and A''. The scale bar represents 15 µm. (B) Hippocampal neurons were double-immunolabeled for GABA_A_ receptors with anti-γ2-subunit antibody (red, upper panel) and with anti-VIAAT antibodies (green, middle panel). Superposition of both immunolabelings (lower panel, merge). Neurons were fixed and immunolabeled at div16. (A) Control cells, non-treated; (A') cultures treated with aminopurvalanol A (5 µM) for 2 days. (A'') cultures treated with aminopurvalanol A (5 µM) for 3 days. Note a higher number of VIAAT-opposed γ2-subunit puncta (yellow, merge) under B, compared to B' and B''. (C) Quantification of the number of gephyrin (mAb7a) puncta. Quantification was done with about 10 cells from 4 independent cultures. Mean ± S.E.; ANOVA with post-hoc test: * P<0.05. (D) Quantification of the number of GABA_A_ receptors with anti-γ2-subunit antibody. Mean ± SE. Quantification was done with 49 cells from n = 4 independent cultures (control), 34 cells from n = 3 independent cultures (2 days) and 18 cells from n = 3 independent cultures (3 days). ANOVA with post-hoc test: ** P<0.01.

To confirm these conclusions in a different experimental approach and to demonstrate that CDKs' enzyme activity is involved, we inhibited CDK enzymes using the antagonists aminopurvalanol A (highest inhibitory activity against CDK 5) [22] or SU9516 (highest inhibitory activity against CDK 5) [22]. We inhibited CDKs for two or three days with 5 µM aminopurvalanol A or SU9516 in hippocampal neurons. As shown in Fig. 4, under these experimental conditions the number of gephyrin clusters detected with mAb7a was reduced from 17.6±2.28 puncta/30 µm dendrite (mean± SE) to 2.91±1.53 puncta/30 µm and 1.0±0.62 puncta/30 µm upon 2 or 3 days inhibition, respectively. Similarly, GABA_A_ receptor γ2 puncta were reduced from 10,9±0.97 puncta/30 µm dendrite (mean ± SE) to 4.33±0.96 puncta/30 µm dendrite (Fig. 4B, 4C) after three days treatment with 5 µM aminopurvalanol A. Similar results were obtained with SU9516 inhibition (data not shown). These results support the conclusions from our CDK5 knockdown experiments, suggesting that CDKs' enzyme activity is crucial for gephyrin phosphorylation and clustering of γ2-containing GABA_A_ receptors in hippocampal neurons.

### Gephyrin phosphorylation is dependent on PP1/PP2a phosphatases

**Figure 5:**
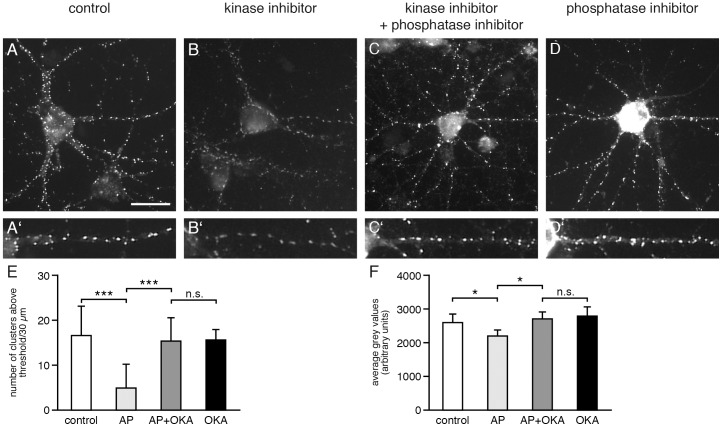
Gephyrin is dephosphorylated at Ser270 by PP1/PP2A phosphatase. (A) Hippocampal neurons were immunolabeled for gephyrin with antibody mAb7a, detecting gephyrin phosphorylation at S270. Neurons were fixed and immunolabeled at div14. (A) Control cells, non-treated; (B) cultures treated with aminopurvalanol A (5 µM) for 6 hours hours; (C) cultures treated with aminopurvalanol A (5 µM) and okadaic acid (250 nM nM) for 6 hours hours; (D) cultures treated with okadaic acid (250 nM nM) for 6 hours. Note a bright mAb7a immunostaining in D, revealing that gephyrin which is present in the soma seems to be dephosphorylated under physiological conditions. The scale bar represents 30 hours. Note a bright mAb7a immunostaining in D, revealing that gephyrin which is present in the soma seems to be dephosphorylated under physiological conditions. The scale bar represents 30 µm. (A'–D') Enlargement of selected dendritic areas shown in A–D. (E) Quantification of the number of gephyrin (mAb7a) puncta above a certain threshold. Quantification was done with 15 cells from 3 independent cultures. Mean ± SE. ANOVA with post-hoc test: *** P<0.001. (F) Quantification of the fluorescence intensity (average gray value) of those puncta above a certain threshold measured in E. Quantification was done with 15 cells from 3 independent cultures. Mean ± SE. ANOVA with post-hoc test: * P<0.05.

To investigate the removal of gephyrin phosphorylation seen upon kinase inhibition we performed experiments inhibiting PP1/PP2a phosphatases known to be involved in the regulation of phosphorylation of gephyrin [23]. As shown in Fig. 5A and 5B, the supplementation of the cell culture medium with 5 µM aminopurvalanol A for six hours before cell fixation reduced mAb7a immunoreactivity. As shown in Fig. 5B and 5E, the number of gephyrin clusters detected with mAb7a above a certain intensity threshold was reduced from 16.4±1.70 puncta/30 µm dendrite (mean± SE) to 4.87±1.36 puncta/30 µm upon six hours inhibition. However, the simultaneous inhibition of CDKs with amimnopurvalanol A (5 µM) and of PP1/PP2a phosphatases with okadaic acid (0.25 µM) for six hours abolished the reduction of mAb7a immunoreactivity observed by inhibiting CDK activity only (Fig. 5C, E). The inhibition of PP1/PP2a phosphatases with okadaic acid (0,25 µM) without kinase inhibition (six hours) resulted in an increase of somatic mAb7a immunoreactivity (Fig. 5D). However, alterations of presynaptic VIAAT staining were obvious (data not shown). Thus, these results indicate that the reduction of mAb7a immunoreactivity observed upon CDK inhibition is due to the activity of PP1/PP2a phosphatases, suggesting that a given level of mAb7a immunoreactivity seen at inhibitory synapses or in the cell soma is the result of a certain balance of CDK kinases and PP1/PP2a phosphatase activities.

### The phosphorylation of gephyrin is cell compartment specific

**Figure 6:**
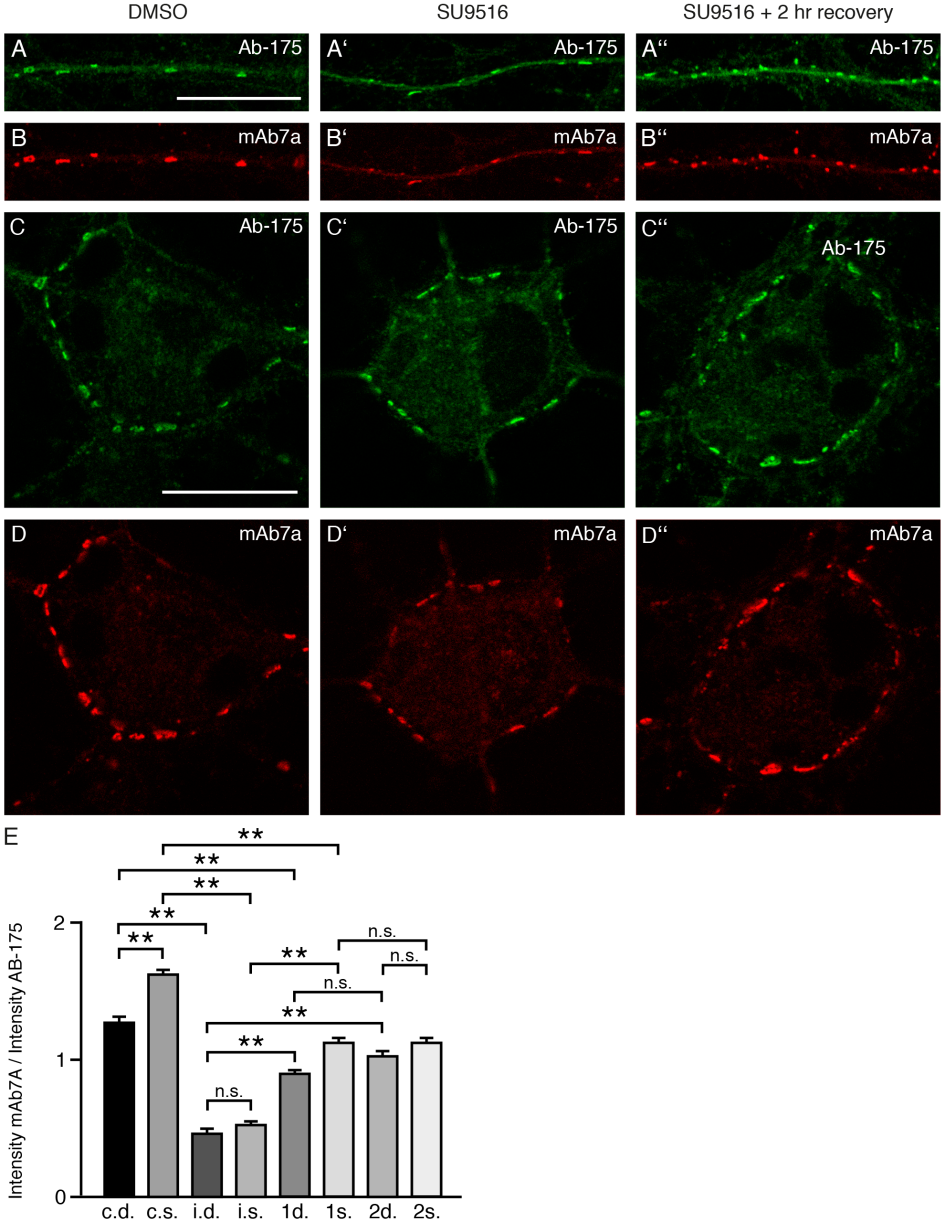
S270 phosphorylation is different at different synapses. Relative fluorescence intensities of double labelling experiments using the pan-gephyrin antibody Ab-175 and the phospho-specific antibody mAb7a were measured in focal planes of confocal microscopy images. (A) Focal plane showing dendritic gephyrin clusters detected with Ab-175 or (B) with the phospho-specific mAb7a antibody. The scale bar represents 10 µm. (A′, B′, E) Upon treatment with CDK5 inhibitor SU9516 (5 µM) for six hours the phosphorylation of gephyrin is significantly reduced. (A′′) After replacement of medium with SU9516 by conditioned medium without inhibitor, cells were fixed and stained after a “recovery” time of two hours. Note increasing phosphorylation as indicated by mAb7a staining seen in B′′ compared to B′. The same analysis in C, C′, C′′ and D′, D′′, D′′ for “somatic” gephyrin clusters, revealing decrease and increase of gephyrin phosphorylation upon SU9516 treatment and “recovery”, respectively (D′′ compared to D′). (E) Quantification of mAb7a-specific phosphorylation comparing the fluorescence intensities of single clusters stained with mAb7a and Ab175 (ratios). **c.d**.: control dendritic clusters; **c.s**.: control somatic clusters; i.d.: CDK5 inhibitor treatment for 6 h, dentritic clusters h, dentritic clusters; i.s.: CDK5 inhibitor treatment for 6 h, somatic clusters h, somatic clusters; **1d**: 1 hour recovery from inhibition, dendritic clusters hour recovery from inhibition, dendritic clusters; **1s**: 1 hour recovery from inhibition, somatic clusters hour recovery from inhibition, somatic clusters; **2d**: 2 hour recovery from inhibition, dendritic clusters hour recovery from inhibition, dendritic clusters; **2s**: 2 hour recovery from inhibition, somatic clusters. Quantification was done with 660 clusters (total) from nine cells from three (n = 3) independent cultures. Mean ± SE. ANOVA with post-hoc test: ** P<0.01.

In order to study the level of mAb7a immunoreactivity and thus the phosphorylation of S270 more directly, we performed CDK activity recovery experiments. For this, we inhibited CDK5 with 5 µM SU9516 for 6 hrs, replaced the culture medium by conditioned medium from sister cultures to terminate CDK inhibition, thus allowing a putative increase of gephyrin phosphorylation for 1 or 2 hours. The incubation of cells was stopped by fixation and cells were double stained for gephyrin with Ab-175 and mAb7a. Next, relative protein levels of somatic and dendritic gephyrin clusters were estimated by quantification of Ab-175 immunofluorescence intensities in focal sections of stained cells by confocal microscopy. The same focus plane and cluster regions were also analyzed for relative fluorescence intensities of mAb7a immunofluorescence (Fig. 6 A–D). The ratio of mAb7a intensities compared to Ab-175 intensities were calculated for each cluster and a total of about 660 gephyrin clusters (52 to 118 for the single conditions) of nine cells from three different cell cultures were analyzed. The results indicate that the relative amount of gephyrin phosphorylation at different gephyrin clusters is different. Interestingly mAb7a/Ab175 relative fluorescence intensities were not randomly distributed from somatic to dendritic gephyrin clusters. However, the relative phosphorylation of gephyrin clusters revealed to be higher at somatic clusters compared to dendritic clusters (Fig. 6E). The comparison of the mAb7a/Ab175 ratio after SU9516 treatment showed a robust reduction of phosphorylation. After medium exchange and incubation of cells in an incubator for one or two hours, kinase activity leading to an increase of mAb7a immunoreactivity was clearly evident, however, it did not reach the level of control cells (Fig. 6E). Thus, the level of gephyrin phosphorylation by CDK5 might be under distinct regulation in different subcellular compartments.

### Gephyrin is phosphorylated by CDK5 at serine residue S270 in vitro

**Figure 7:**
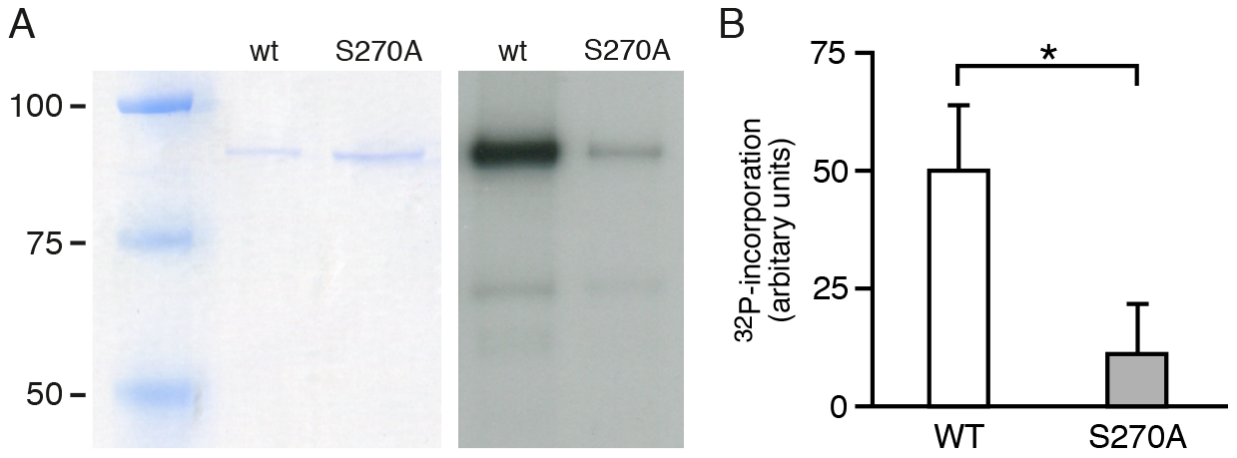
Substitution of S270 by alanine reduces phosphorylation of gephyrin by CDK5/p25. (A) Coomassie blue staining of SDS-PAGE gels (left panel) to control for equal input amounts of wild type 6xHis-gephyrin (wt) and S270A mutant proteins and representative autoradiogram (right panel) of wt and S270A mutant proteins subjected to *in vitro* kinase assay with recombinant CDK5/p25 in the presence of [γ-^32^P] ATP and separated with SDS-PAGE. (B) Quantification of band intensities of autoradiograms shown in A of three independent experiments (n = 3). Mean ± SD. Student's t-test, *P<0.05.

Previous work has shown that gephyrin can be phosphorylated *in vitro* at serine and/or threonine residues by endogenous protein kinases [24]. Moreover, S270 was identified as one putative phosphorylation site sensitive to the phospho-specific antibody mAb7a [15]. To verify whether S270 of gephyrin can serve as a CDK5 phosphorylation site, purified 6xHis-tagged wild type and mutant gephyrin^S270A^ was subjected to *in vitro* kinase assays using CDK5/p25 and [γ-^32^P] ATP. Wild type gephyrin was labeled strongly with ^32^P after incubation with CDK5/p25 (Fig. 7). Importantly, gephyrin mutant gephyrin^S270A^ revealed a robust reduction in ^32^P-incorporation (Fig. 7A, B), suggesting that this site is phosphorylated by CDK5 in wild type gephyrin.

## Discussion

We report the following major findings: First, CDK5 activity is involved in the phosphorylation of gephyrin detected with the phospho-specific antibody mAb7a. Second, CDK5 is important for the stability of GABA_A_ receptor clusters in hippocampal neurons. Third, gephyrin is dephosphorylated by PP1/PP2A antagonizing the CDK5-dependent phosphorylation. Fourth, the relative level of phosphorylation detected with mAb7a is cell compartment-specific. Finally, gephyrin can be phosphorylated by CDK5 at position S270 *in vitro*. The phosphorylation of this site is strongly reduced upon CDK5 knockdown and pharmacological inhibition.

Pharmacological inhibition of cyclin-dependent kinases including CDK5, −2 and −1 in hippocampal neurons for 1- to 24 hours reduced postsynaptic gephyrin phosphorylation at S270 [15]. Therefore, we used RNA interference to target CDK5, as one candidate kinase, and evaluated its effect on the phosphorylation of gephyrin and on clustering of GABA_A_ receptors.

Cultured hippocampal neurons infected with CDK5 knockdown constructs displayed an average decrease of 50% of CDK5 immunoreactivity compared to neighboring non-infected neurons. Quantification of gephyrin clusters detected with the phospho-specific antibody mAb7a revealed that the CDK5 knockdown with three different CDK5-specific shRNAs resulted in a drastic reduction of the number of mAb7a immunoreactive puncta (Fig. 1C). Importantly, also the number and fluorescence intensity of γ2 containing GABA_A_ receptor puncta were reduced (Fig. 2). These results suggest that CDK5 plays a functional role at inhibitory synapses of hippocampal neurons. This conclusion was supported by other experiments using prolonged pharmacological inhibition with aminopurvalanol A and SU9516 revealing that inhibition of kinase activity resulted in a reduction of mAb7a and γ2 containing GABA_A_ receptor puncta. These results showed for the first time that a reduced phosphorylation of gephyrin at position S270 coincides with a reduction of postsynaptic ion channels after prolonged inhibition of two to three days, suggesting that the reduced gephyrin phosphorylation might be functionally linked to reduced GABA_A_ receptor cluster stability. However, also indirect effects of prolonged kinase inhibition can not be excluded.

Reduced phosphorylation of gephyrin upon CDK5 knockdown or inhibition is most likely due to the dephosphorylation of gephyrin by PP1/PP2A, because simultaneous inhibition of these phosphatases and CDKs abolished the reduction of phosphorylation seen with CDK inhibition only. These results may help to resolve the discrepancy in the literature concerning the role of gephyrin phosphorylation for cluster formation relying on experiments using only phosphatase inhibition. For to resolve the functional implication of phosphorylation for gephyrin cluster formation it might be necessary to study both kinase and phosphatase functions together.

Ablation of gephyrin expression in mice causes the loss of synaptically localized GlyR and GABA_A_ receptors containing α2 and γ2 subunits [25],[26]. Consistent with this, the reduction of mAb7a gephyrin puncta upon CDK5 knockdown or inhibition with aminopurvalanol A or SU9516 for three days coincides with a reduced number of γ2 subunit-containing GABA_A_ receptor clusters at postsynaptic compartments. Thus, phosphorylation of gephyrin or other substrates by CDK5 may be one molecular mechanism determining GABA_A_ receptor cluster stability in hippocampal neurons. Consistent with this interpretation are the results from former studies [15], [27], where we could show that knockdown of the gephyrin-binding protein collybistin in hippocampal neurons abolishes gephyrin phosphorylation detected with mAb7a [15], and resulted in a robust reduction of functional postsynaptic GABA_A_ receptors containing γ2 subunits [27].

These results are in agreement with the current model of gephyrin and receptor clustering. It was postulated that at postsynaptic membrane specializations gephyrin forms a regular hexagonal lattice by trimerization of the *N*-terminal domain (G-domain) and dimerization of the carboxy-terminal domain (E-domain) [28]. Specific gephyrin conformations, posttranslational-modifications and the interaction with specific proteins such as collybistin are thought to be the main determinants of gephyrin and receptor clustering at inhibitory postsynapses [29], [30].

To verify whether gephyrin serves as a CDK5 substrate, gephyrin and a gephyrin mutant were subjected to *in vitro* kinase assays using CDK5/p25 and [γ-^32^P] ATP. Consistent with earlier findings [15], gephyrin phosphorylation by CDK5/p25 could be demonstrated at amino acid position S270. According to these observations, the loss of mAb7a immunoreactivity upon CDK5 knockdown indicates most likely a reduced phosphorylation at S270. However, the presence of additional CDK5-dependent phosphorylation sites within gephyrin is predicted [31]. Thus, we hypothesize that CDK5 might phosphorylate S270 and additional unknown sites, which, together might determine gephyrin conformation, gephyrin protein interactions and gephyrin cluster formation and stability. For example, gephyrin proline-directed phosphorylation at S188, S194 and S200 was shown to be important for the recruitment of the peptidyl-prolyl isomerase Pin1. The deletion of the entire proline-rich domain or point mutations at S188, S194 and S200 residues almost completely ablated Pin1 binding to gephyrin and Pin1^-/-^ mice revealed reduced glycine receptor cluster numbers [32].

Other studies have also shown that gephyrin is phosphorylated on several serine and threonine residues, mostly in the central region (C-domain) [31]–[34]. In particular GSK3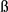 was shown to phosphorylate S270, thereby decreasing the number of gephyrin clusters [33]. In addition, a neighboring serine at position 268 was also shown to reduce cluster formation upon phosphorylation by ERK1 [34]. One possible explanation for these discrepancies could be that these sites might be phosphorylated by different kinases and a specific combinatorial pattern of phosphorylations might be have divergent functional consequences. Moreover, GSK3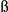-mediated phosphorylation of gephyrin might be primed by CDK5, thus explaining that the phosphorylation at S270 is dependent on both, CDK5 and GSK3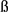 activity.

Posttranslational modification of receptor subunits is another mechanism to determine the formation of postsynaptic assemblies of ligand-gated ion channels and scaffold proteins. For example, protein kinase C was shown to regulate the glycine receptor (GlyR) diffusion properties and gephyrin interaction by phosphorylating residue S403 of the β-subunit of the GlyR [35]. The membrane insertion and turnover of different GABA_A_ receptor subunits are regulated by phosphorylation of subunits [36]. For example, S383 within the β3 subunit is phosphorylated by Ca^2+^ calmodulin- dependent protein kinase II, resulting in a rapid insertion of GABA_A_ receptors in the cell membrane [37]. Moreover, GABA_A_ receptor γ subunits are phosphorylated at Y365 and Y367, reducing receptor internalization and increasing tonic inhibition [38]. Thus, CDK5-dependent alteration of receptor subunit phosphorylation might contribute to GABA_A_ receptor cluster formation or stability independent of gephyrin phosphorylation.

In spinal cord neurons, gephyrin clustering is regulated by GlyR activity [39]. More recently, integrin signaling was identified to determine GlyR and gephyrin trafficking at synapses [40]. In addition, BDNF receptor signaling was shown to determine gephyrin clustering [41]. Thus, these surface signaling molecules might be active upstream of CDK5 [9] as a putative regulator of gephyrin and/or GABA_A_ receptor clustering at inhibitory synapses.

## References

[1] Meyerson M, Enders GH, Wu C, Su LK, Gorka C, et al (1992) A family of human cdc2-related protein kinases. EMBO J 11: 2909–2017. 1639063 10.1002/j.1460-2075.1992.tb05360.x PMC556772

[2] Lew J, Huang QQ, Qi Z, Winkfein RJ, Aebersold R, et al (1994) A brain-specific activator of cyclin-dependent kinase 5. Nature 371: 423–426. 8090222 10.1038/371423a0

[3] Tsai LH, Delalle I, Caviness VS Jr, Chae T, Harlow E (1994) p35 is a neural-specific regulatory subunit of cyclin-dependent kinase 5. Nature 371: 419–423. 8090221 10.1038/371419a0

[4] Patrick GN, Zukerberg L, Nikolic M, de la Monte S, Dikkes P, et al (1999) Conversion of p35 to p25 deregulates CDK5 activity and promotes neurodegeneration. Nature 402: 615–622. 10604467 10.1038/45159

[5] Cheung ZH, Ip NY (2011) CDK5: a multifaceted kinase in neurodegenerative diseases. Trends in Cell Biol 22: 169–175. 22189166 10.1016/j.tcb.2011.11.003

[6] Hirasawa M, Ohshima T, Takahashi S, Longenecker G, Honjo Y, et al (2004) Perinatal abrogation of Cdk5 expression in brain results in neuronal migration defects. Proc Natl Acad Sci USA 101: 6249–6254. 15067135 10.1073/pnas.0307322101 PMC395955

[7] Ohshima T, Ward JM, Huh CG, Longenecker G, Veeranna Pant HC, et al (1996) Targeted disruption of the cyclin-dependent kinase 5 gene results in abnormal corticogenesis, neuronal pathology and perinatal death. Proc Natl Acad Sci USA 93: 11173–11178. 8855328 10.1073/pnas.93.20.11173 PMC38303

[8] Zukerberg LR, Patrick GN, Nikolic M, Humbert S, Wu CL, et al (2000) Cables links CDK5 and c-Abl and facilitates CDK5 tyrosine phosphorylation, kinase upregulation, and neurite outgrowth. Neuron 26: 633–646. 10896159 10.1016/s0896-6273(00)81200-3

[9] Cheung ZH, Chin WH, Chen Y, Ng YP, Ip NY (2007) CDK5 is involved in BDNF-stimulated dendritic growth in hippocampal neurons. PLoS Biol 5: e63. 17341134 10.1371/journal.pbio.0050063 PMC1808488

[10] Ou CY, Poon VY, Maeder CI, Watanabe S, Lehrman EK, et al (2011) Two cyclin-dependent kinase pathways are essential for polarized trafficking of presynaptic components. Cell 141: 846–858. 10.1016/j.cell.2010.04.011 PMC3168554 20510931

[11] Morabito MA, Sheng M, Tsai LH (2004) Cyclin-dependent kinase 5 phosphorylates the N-terminal domain of the postsynaptic density protein PSD-95 in neurons. J Neurosci 24: 865–876. 14749431 10.1523/JNEUROSCI.4582-03.2004 PMC6729809

[12] Zhang S, Edelmann L, Liu J, Crandall JE, Morabito MA (2008) CDK5 regulates the phosphorylation of tyrosine 1472 NR2B and the surface expression of NMDA receptors. J Neurosci 28: 415–424. 18184784 10.1523/JNEUROSCI.1900-07.2008 PMC6670547

[13] Li BS, Sun MK, Zhang L, Takahashi S, Ma W, et al (2001) Regulation of NMDA receptors by cyclin-dependent kinase-5. Proc Natl Acad Sci USA 98: 12742–12747. 11675505 10.1073/pnas.211428098 PMC60124

[14] Guan JS, Su SC, Gao J, Joseph N, Xie Z, et al (2011) Cdk5 Is Required for Memory Function and Hippocampal Plasticity via the cAMP Signaling Pathway. PLoS One 6, e25735. 21984943 10.1371/journal.pone.0025735 PMC3184170

[15] Kuhse J, Kalbouneh H, Schlicksupp A, Mükusch S, Nawrotzki R, et al (2012) Phosphorylation of gephyrin in hippocampal neurons by cyclin-dependent kinase CDK5 at Ser-270 is dependent on collybistin. J Biol Chem 287: 30952–30966. 22778260 10.1074/jbc.M112.349597 PMC3438928

[16] Lois C, Hong E, Pease S, Brown EJ, Baltimore D (2002) Germline transmission and tissue-specific expression of transgenes delivered by lentiviral vectors. Science 295: 868–872. 11786607 10.1126/science.1067081

[17] Dresbach T, Hempelmann A, Spilker C, Tomdieck S, Altrock WD, et al (2003) Functional regions of the presynaptic cytomatrix protein bassoon: significance for synaptic targeting and cytomatrix anchoring. Mol Cell Neurosci 23: 279–291. 12812759 10.1016/s1044-7431(03)00015-0

[18] Pfeiffer F, Simler R, Grenningloh G, Betz H (1984) Monoclonal antibodies and peptide mapping reveal structural similarities between the subunits of the glycine receptor of rat spinal cord. Proc Natl Acad Sci USA 81: 7224–7227. 6095276 10.1073/pnas.81.22.7224 PMC392111

[19] Nawrotzki R, Islinger M, Vogel I, Völkl A, Kirsch J (2012) Expression and subcellular distribution of gephyrin in non-neuronal tissues and cells. Histochem Cell Biol 137: 471–482. 22270318 10.1007/s00418-012-0914-7

[20] Essrich C, Lorez M, Benson JA, Fritschy JM, Lüscher B (1998) Postsynaptic clustering of major GABA_A_ receptor subtypes requires the gamma 2 subunit and gephyrin. Nature Neurosci 1: 563–571. 10196563 10.1038/2798

[21] Kneussel M, Brandstatter JH, Gasnier B, Feng G, Sanes JR, et al (2001) Gephyrin-independent clustering of postsynaptic GABA (A) receptor subtypes. Mol Cell Neurosci 17: 973–982. 11414787 10.1006/mcne.2001.0983

[22] Pawson AJ, Sharman JL, Benson HE, Faccenda E, Alexander SPH, et al (2014) The IUPHAR/BPS Guide to PHARMACOLOGY: an expert-driven knowledgebase of drug targets and their ligands. Nucleic Acids Res 42(D1): D1098–D1106. 24234439 10.1093/nar/gkt1143 PMC3965070

[23] Bausen M, Weltzien F, Betz H, O'Sullivan GA (2010) Regulation of postsynaptic gephyrin cluster size by protein phosphatase 1. Mol Cell Neurosci 44: 201–209. 20206270 10.1016/j.mcn.2010.02.007

[24] Langosch D, Hoch W, Betz H (1992) The 93 kDa protein gephyrin and tubulin associated with the inhibitory glycine receptor are phosphorylated by an endogenous protein kinase. FEBS L 298: 113–117. 10.1016/0014-5793(92)80034-e 1312018

[25] Feng G, Tintrup H, Kirsch J, Nichol MC, Kuhse J, et al (1998) Dual requirement for gephyrin in glycine receptor clustering and molybdoenzyme activity. Science 282: 1321–1324. 9812897 10.1126/science.282.5392.1321

[26] Kneussel M, Betz H (2000) Receptors, gephyrin and gephyrin-associated proteins: novel insights into the assembly of inhibitory postsynaptic membrane specializations. J Physiol 525: 1–9. 10811719 10.1111/j.1469-7793.2000.t01-4-00001.x PMC2269938

[27] Körber C, Richter A, Kaiser M, Schlicksupp A, Mükusch S, et al (2012) Effects of distinct collybistin isoforms on the formation of GABAergic synapses in hippocampal neurons. Mol Cell Neurosci 50: 250–259. 22659578 10.1016/j.mcn.2012.05.006

[28] Saiyed T, Paarmann I, Schmitt B, Haeger S, Sola M, et al (2007) Molecular basis of gephyrin clustering at inhibitory synapses: role of G- and E-domain interactions. J Biol Chem 282: 5625–5632. 17182610 10.1074/jbc.M610290200

[29] Kins S, Betz H, Kirsch J (2000) Collybistin, a newly identified brain-specific GEF, induces submembrane clustering of gephyrin. Nat Neurosci 3: 22–29. 10607391 10.1038/71096

[30] Harvey K, Duguid IC, Alldred MJ, Beatty SE, Ward H, et al (2004) The GDP-GTP exchange factor collybistin: an essential determinant of neuronal gephyrin clustering. J Neurosci 24: 5816–5826. 15215304 10.1523/JNEUROSCI.1184-04.2004 PMC6729214

[31] Herweg J, Schwarz G (2012) Splice-specific glycine receptor binding, folding, and phosphorylation of the scaffolding protein gephyrin. J. Biol. Chem 287: 12645–12656. 22351777 10.1074/jbc.M112.341826 PMC3339950

[32] Zita MM, Marchionni I, Bottos E, Righi M, Del Sal G, et al (2007) Post-phosphorylation prolyl isomerisation of gephyrin represents a mechanism to modulate glycine receptors function. EMBO J 26: 1761–1771. 17347650 10.1038/sj.emboj.7601625 PMC1847658

[33] Tyagarajan SK, Ghosh H, Yévenes GE, Nikonenko I, Ebeling C, et al (2010) Regulation of GABAergic synapse formation and plasticity by GSK3beta-dependent phosphorylation of gephyrin. Proc Natl Acad Sci USA 108: 379–384. 21173228 10.1073/pnas.1011824108 PMC3017200

[34] Tyagarajan SK, Ghosh H, Yevenes GE, Imanishi SY, Zeilhofer HU, et al (2013) ERK and GSK3beta regulate gephyrin postsynaptic aggregation and GABAergic synaptic function in a calpain-dependent mechanism. J Biol Chem 288: 9634–9647. 23408424 10.1074/jbc.M112.442616 PMC3617267

[35] Specht CG, Grunewald N, Pascual O, Rostgaard N, Schwarz G, et al (2011) Regulation of glycine receptor diffusion properties and gephyrin interactions by protein kinase C. EMBO J 30: 3842–3853. 21829170 10.1038/emboj.2011.276 PMC3173796

[36] Vithlani ML, Moss SJ (2009) The role of GABAAR phosphorylation in the construction of inhibitory synapses and the efficacy of neuronal inhibition. Biochem Soc Trans 37: 1355–1358. 19909275 10.1042/BST0371355 PMC2846645

[37] Saliba RS, Kretschmannova K, Moss SJ (2012) Activity-dependent phosphorylation of GABAA receptors regulates receptor insertion and tonic current. EMBO J 31: 2937–2951. 22531784 10.1038/emboj.2012.109 PMC3395084

[38] Nani FL, Bright DP, Revilla-Sanchez R, Tretter V, Moss SJ, et al (2013) Tyrosine phosphorylation of GABAA receptor γ2-subunit regulates tonic and phasic inhibition in the thalamus. J Neurosci 33: 12718–12727. 23904608 10.1523/JNEUROSCI.0388-13.2013 PMC4400286

[39] Kirsch J, Betz H (1998) Glycine-receptor activation is required for receptor clustering in spinal neurons. Nature 392: 717–720. 9565032 10.1038/33694

[40] Charrier C, Machado P, Tweedie-Cullen RY, Rutishauser D, Mansuy IM, et al (2010) A crosstalk between β1 and β3 integrins controls glycine receptor and gephyrin trafficking at synapses. Nat Neurosci 13: 1388–1395. 20935643 10.1038/nn.2645

[41] Wuchter J, Beuter S, Treindl F, Herrmann T, Zeck G, et al (2012) A comprehensive small interfering RNA screen identifies signaling pathways required for gephyrin clustering. J Neurosci 32: 14821–14834. 23077067 10.1523/JNEUROSCI.1261-12.2012 PMC6621453

